# Considerations for clinical read alignment and mutational profiling using next-generation sequencing

**DOI:** 10.12688/f1000research.1-2.v2

**Published:** 2012-09-20

**Authors:** Gavin R Oliver

**Affiliations:** 1 ALMAC, Craigavon, Co. Armagh, UK

## Abstract

Next-generation sequencing technologies are increasingly being applied in clinical settings, however the data are characterized by a range of platform-specific artifacts making downstream analysis problematic and error- prone. One major application of NGS is in the profiling of clinically relevant mutations whereby sequences are aligned to a reference genome and potential mutations assessed and scored. Accurate sequence alignment is pivotal in reliable assessment of potential mutations however selection of appropriate alignment tools is a non-trivial task complicated by the availability of multiple solutions each with its own performance characteristics. Using targeted analysis of BRCA1 as an example, we have simulated and mutated a test dataset based on Illumina sequencing technology. Our findings reveal key differences in the abilities of a range of common commercial and open source alignment tools to facilitate accurate downstream detection of a range of mutations. These observations will be of importance to anyone using NGS to profile mutations in clinical or basic research.

## Introduction

Since emergence in 2005, next-generation sequencing (NGS) technologies have proven prolific tools in the research setting, permeating a variety of scientific disciplines and demonstrating a range of applications that seems to be limited only by the imagination of the sequencing community. The technology continues to develop at a rapid pace with established instrument manufacturers regularly augmenting their product portfolios and an increasing number of start-up companies promising to disrupt the market. Beyond basic research applications, NGS technologies are now increasingly being applied in the clinical environment, driven partly by their rapid maturation and the arrival to market of smaller, cheaper sequencing platforms.

The potential clinical application of NGS has a broad scope ranging from full human genome profiling ^1^ to investigation of the microbiome ^2^ and includes applications such as biomarker discovery, patient diagnosis, prediction of drug response and patient stratification for clinical trials. Such applications often involve the targeted profiling of genes known to be of clinical relevance. These genes harbor diagnostically relevant variants including single nucleotide polymorphisms (SNPs), and small insertions and deletions (INDELs). Individual genes have previously been interrogated in clinical testing using traditional techniques such as Sanger sequencing however NGS technologies have already begun to supplant the previous tools of choice in these areas, offering increased speed and throughput with reduced running costs.

Despite many successes and increasing uptake, the data generated by NGS analyzers is not perfect, with each platform yielding characteristic errors and biases. Furthermore, NGS technologies produce reads that are much shorter than those traditionally produced by Sanger sequencing methods and this can complicate matters further, especially in genomes containing a large proportion of repetitive elements ^3^. The effect of these problems is most visible in large scale studies such as genome-wide sequencing where a recent study reported a 1 million variant platform-based discrepancy for a single genome ^4^. This fact bestows responsibility on both algorithm and software developers and downstream users to develop deep understanding of the various data types and their idiosyncrasies and to apply this appreciation in their analysis and interpretation, in order to correct or compensate for potential errors. Despite forming an area of active research, data interpretation remains an issue and is no doubt a factor in feeding the inertia of many clinical facilities that are reluctant to adopt the new technologies ^5^.

Two major computational steps in variant detection from NGS data are read alignment whereby the data are mapped to corresponding locations on a target genome, and mutation calling whereby nucleotides differing from the target genome are assessed and scored on their likelihood of representing a genuine mutation versus an error. While these two stages of analysis may be supplemented with various pre or post processing techniques they represent the most crucial steps and therefore the area of most active software and algorithm development.

Aligners of choice have begun to emerge ^6, 7^ however their strengths are often application specific and different tools are recommended depending on the sequencing platform and individual study goals. Often there is a trade-off between speed and sensitivity at the read alignment stage with speed sometimes prioritized due to the volumes of data produced by NGS technologies and the corresponding time required for analysis. Aligners can be classified as gapped or ungapped based on their ability to produce successful alignments in the presence of small INDELs. Aligners including BWA ^8^ and Stampy have been shown to produce alignments with a fair degree of success for reads containing a range of INDEL sizes ^9^ however such abilities will vary based on a range of complicating factors including size and location of the INDEL. As well as generating continuous controversy ^10^, the *BRCA1* gene presents a particularly interesting set of alignment challenges due to a disproportionately high concentration of INDELs greater than eight nucleotides in length. In fact, 3% of known deleterious mutations in the Breast Cancer Information Core (BIC) database ^11^ fall into this category and represent a significant over-representation when compared to a healthy genome. Furthermore the *BRCA1* gene contains multiple areas of high shared identity in the form of tandem repeats, posing another difficulty in achieving accurate read mapping. Challenges like this pose particular difficulties in the clinical setting where errors have the potential to translate to misdiagnosis or mistreatment, directly affecting and endangering the lives of patients. No gold-standard clinical alignment tools yet exist and numerous publicized examples of early translational work appear to base their choice of tool on user-friendliness or availability of a graphical-user-interface rather than assessments of performance. We have investigated the performance of a range of popular alignment tools and assessed their ability to facilitate accurate downstream detection of known mutations with a commonly used variant calling pipeline. Several of these tools are already being used as components of diagnostic workflows in the clinical setting. Here we present data generated using *BRCA1* reads created *in-silico* in a simulated, targeted sequencing scenario. Our findings demonstrate the widely varying abilities of common read alignment tools and their impact on downstream variant calling. Furthermore the results suggest a need for careful and thorough evaluation of the tools used in a particular analysis pipeline by simulation and analysis of data of known constitution.

## Methods

### Aligners

A range of open source and commercial alignment tools were selected for assessment based on their reported ability to facilitate detection of both SNPs and INDELs as well as frequency of citation in both scientific and commercial literature. The aligners included in the comparison were: BWA (0.5.9-r16), Bfast (0.7.0a) ^12^, Smalt (0.5.8), Stampy (1.0.14), Mosaik (2.1.33), CLC Genomics Workbench’s (5.0.1) NGS and Beta aligner, Novocraft’s Novoalign (V2.07.18) ^13^, Omixon’s Variant Toolkit (2.1.3), Bowtie 2 (2.0.0-beta5) ^14^ and Softgenetics Nextgene (2.2) aligner.

### Read simulation

Stampy was used to simulate sixty-seven groups of 200,000 90bp paired-end FASTQ Illumina reads from the human *BRCA1* gene (hg19) with an appropriate error profile. Each sequence grouping was mutated *in-silico* with custom scripts used to introduce a combination of 20 SNPs and 13 INDELs from a test set of 2211 (1299 unique) known *BRCA1* variants containing 1340 SNPs, 320 insertions and 551 deletions. The test set was randomly selected from the full collection of *BRCA1* mutations in dbSNP v131 ^15^ and overlapping mutations removed.

Simulated Illumina BRCA1 reads in FASTQ format Sixty-seven groups of 200,000 90bp paired-end FASTQ Illumina reads from the human BRCA1 gene (hg19). Each sequence grouping was mutated in-silico to introduce a combination of 20 SNPs and 13 INDELs.

VCF files describing the known mutations in each read file VCF files describing the mutations contained in the sixty-seven groups of BRCA1 reads. These were selected from a test set of 2211 (1299 unique) known BRCA1 variants containing 1340 SNPs, 320 insertions and 551 deletions.

### Read alignment

Reads were aligned to hg19 chromosome 17 on a HP DL585 G6 server with 4 six-core AMD Opteron 2.8Ghz processors and 256GB of RAM. Multi-threading with the maximum number of threads supported by the aligner was utilized. The commands used to run the aligners are provided for all aligners with the exception of the CLC and NextGene software which are GUI based and were run with default settings. Each aligner was run in both single-end and paired-end mode with half of the paired-end reads being used to simulate a single-end read dataset. Run-times were recorded based on the wall-clock time taken to align and produce a SAM format output for all 67 sets of FASTQ reads (i.e. 13.4 million reads). Index creation was not included as this is a one-off step for any reference.

Aligner execution scripts BASH scripts to process SAM files and perform variant calling with GATK

### Calling of SNPs and INDELs

Each SAM formatted file was converted to BAM format and processed to ensure downstream compatibility with GATK ^16^ using a combination of tools from the Picard collection. (SamFormatConverter, AddOrReplaceReadGroups, SortSam and BuildBamIndex respectively). BAM files were then processed in a GATK-based pipeline. The pipeline consisted of local realignment around INDELs (RealignerTargetCreator and IndelRealigner), quality score recalibration (CountCovariates and TableRecalibrator) and finally variant calling (UnifiedGenotyper). The wrapper scripts sam2bam.sh and gatk.sh are provided and can be used to recreate the processing steps from alignment files (SAM format) to variant call (VCF format).

Alignment processing and variant calling scripts BASH scripts to perform alignment of reads to reference

Aligner-specific VCF files containing the mutations called for each set of reads in single and paired-end modes VCF files describing the mutations detected in the sixty-seven groups of BRCA1 reads when testing each alignment program in paired-end and single-end mode.

## Results/discussion

### Mutation panel

The reads and mutation panel utilized here represent a challenging and multi-functional test set with widely varying INDEL sizes ( Table 1) and an extensive range of SNPs providing a useful means of assessing the aligners’ effects on variant calling in single and paired-end read modes. The reads were created to contain only known mutations from the human *BRCA1* gene thus facilitating downstream assessment of mutation profiling accuracy whilst remaining comparable to real-world data. Reads were simulated to closely match the error profile of Illumina’s sequencing technology enabling a further level of realism to be captured in the simulated test-set. Only homozygous variants at high levels of sequence coverage (70–140x) were included in the test set to focus testing of the alignment tools’ abilities rather than the quality of the downstream variant calling methods.

**Table 1.** Range of INDEL sizes (in base pairs) in the *BRCA1* mutation panel. A representative range of SNPs and small INDELs corresponding to known mutations were distributed between 67 groups of simulated Illumina reads in order to test the aligners’ abilities to facilitate accurate downstream detection of mutations.

Mutation size	# Insertions	# Deletions
1	215	261
2	52	121
3	8	40
4	11	60
5	5	27
6	11	6
7	4	7
8	3	10
9	0	2
11	0	9
12	1	0
13	0	1
14	3	0
15	1	1
16	1	0
17	0	2
18	1	0
19	0	1
24	4	0
29	0	1
62	0	1
64	0	1

### Run-times

Run-times varied widely from seconds to hours Table 2. Novocraft’s Novoalign software performed fastest on this dataset and was closely followed by BWA and Bowtie 2 in both single and paired-end mode whilst Bfast’s paired-end mode represented the slowest run-time by almost half a day. Nextgene was excluded from this comparison due to the fact it is Windows-based software and it was not possible to assess run-times on the same hardware as for the other aligners. For all aligners studied here, alignment time could arguably be considered acceptable for the application simulated i.e. alignment of targeted sequencing reads for 64 samples. However, it should be noted that a typical exome data set can be around around 7 times the 13.4 million reads used here and full genome datasets are larger still. Thus, some of the aligners tested in this targeted sequencing application would likely prove unsuitable for larger scale applications. What constitutes an acceptable running time will vary and should be considered on a per-application basis in association with other performance characteristics. It should also be noted that the ranking of aligners by speed observed here could vary based on the size of the input dataset.

**Table 2.** Aligner run times in wall-clock hours. Each aligner was tested by aligning all 67 groups of simulated Illumina reads in single and paired-end mode. The maximum number of threads utilizable by each aligner were used in testing.

Aligner	# Threads Utilizable (of 24)	PE Time to align	SE Time to align
BWA	24	0.28	0.08
Bfast	24	19.79	7.01
Smalt	8	6.44	8.25
Stampy	1	6.28	3.50
Mosaik	24	2.64	1.56
CLC	24	1.41	0.88
CLCBeta	24	0.67	0.60
Novoalign	24	0.11	0.04
Omixon	24	4.83	1.26
Bowtie2	24	0.30	0.11
Nextgene	24	NA	NA

### Sensitivity of detection

**Figure 1.**
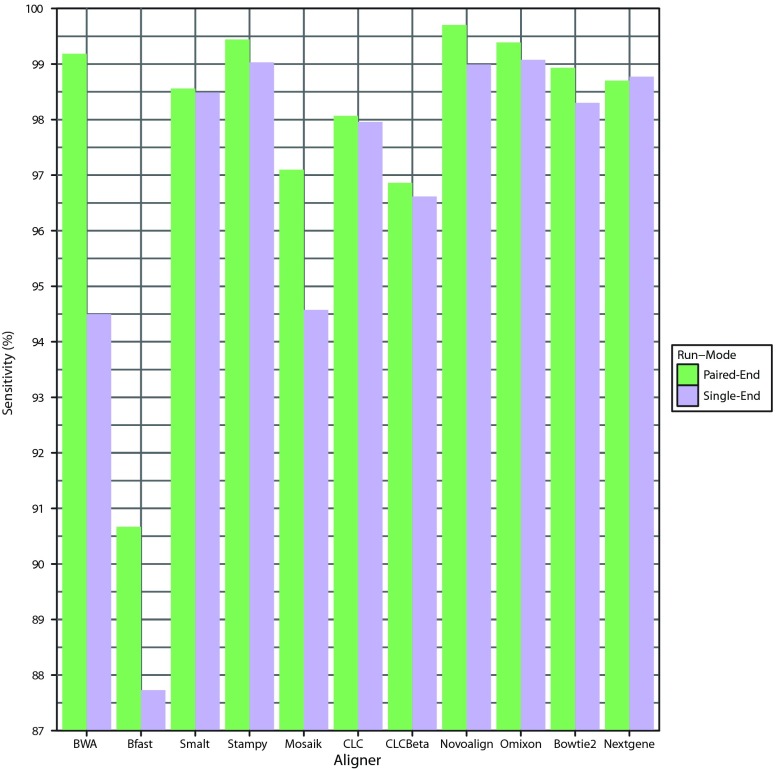
Overall sensitivity of downstream mutation detection. Graphical representation of the total percentage of mutations detected across 67 groups of simulated Illumina reads following alignment in single-end and paired-end mode with each aligner.

The greatest overall sensitivities of detection were achieved following alignment with Novoalign in paired-end mode and Omixon’s Variant Toolkit in single-end mode ( Figure 1, Table 3). Stampy also enabled highly sensitive mutation detection with Bfast performing least favorably. Sensitivities were also assessed based on the category of mutation ( Table 4). Some aligners such as Smalt, Bowtie 2 and CLC were clearly seen to facilitate better detection of SNPs than INDELs in general. Nextgene performed similarly for SNPs and deletions but gave way to lower downstream sensitivities of insertion detection. BWA showed obvious decreases in ability to enable accurate detection of INDELs when moving from paired-end to single-end mode. In contrast, Novoalign, Omixon and Stampy performed well regardless of run-mode or mutation type. Overall Novoalign was the best performer in paired-end mode while Omixon achieved the highest downstream detection sensitivities in single-end mode. In a clinical environment, sensitivity will likely represent the most important metric in evaluating alignment software, however other factors may also be of importance, depending on the test in question. Specificity values are not included here as they were non-discriminatory in this context due to low numbers of false positives relative to the high number of true negatives.

**Table 3.** Overall detection sensitivities. The total percentage of mutations detected post-alignment across the 67 groups of simulated Illumina reads was recorded for single and paired-end run-modes. Each read group contained 13 INDELs of varying sizes as well as 20 SNPs.

Aligner	Sensitivity PE	Sensitivity SE
BWA	99.23	94.53
Bfast	90.68	87.83
Smalt	98.55	98.51
Stampy	99.46	99.05
Mosaik	97.06	95.07
CLC	98.01	97.92
CLCBeta	96.88	96.74
Novoalign	99.59	99.00
Omixon	99.41	99.14
Bowtie2	98.91	98.19
Nextgene	98.64	98.69

**Table 4.** Downstream detection sensitivities by mutation type. Total percentage of SNPs and INDELs detected across the 67 groups of simulated Illumina reads following alignment with each aligner in single and paired-end mode.

Aligner	% SNPs found PE	% SNPs found SE	% Insertions found PE	% Insertions found SE	% Deletions found PE	% Deletions found SE
BWA	99.48	99.48	99.38	89.38	98.55	85.48
Bfast	92.69	89.85	83.13	78.75	90.20	88.20
Smalt	99.40	99.40	96.56	96.25	97.64	97.64
Stampy	99.48	99.25	99.38	98.13	99.46	99.09
Mosaik	97.01	96.34	96.25	90.00	97.64	94.92
CLC	99.40	99.40	96.56	96.25	95.46	95.28
CLCBeta	97.61	97.61	95.31	95.00	96.01	95.64
Novoalign	99.70	99.48	100.00	98.13	99.09	98.37
Omixon	99.40	98.96	99.38	99.38	99.46	99.46
Bowtie2	99.50	99.50	97.50	96.60	98.40	96.00
Nextgene	99.00	99.00	96.60	96.60	99.10	99.10

### Incorrect identification of mutations

**Figure 2.**
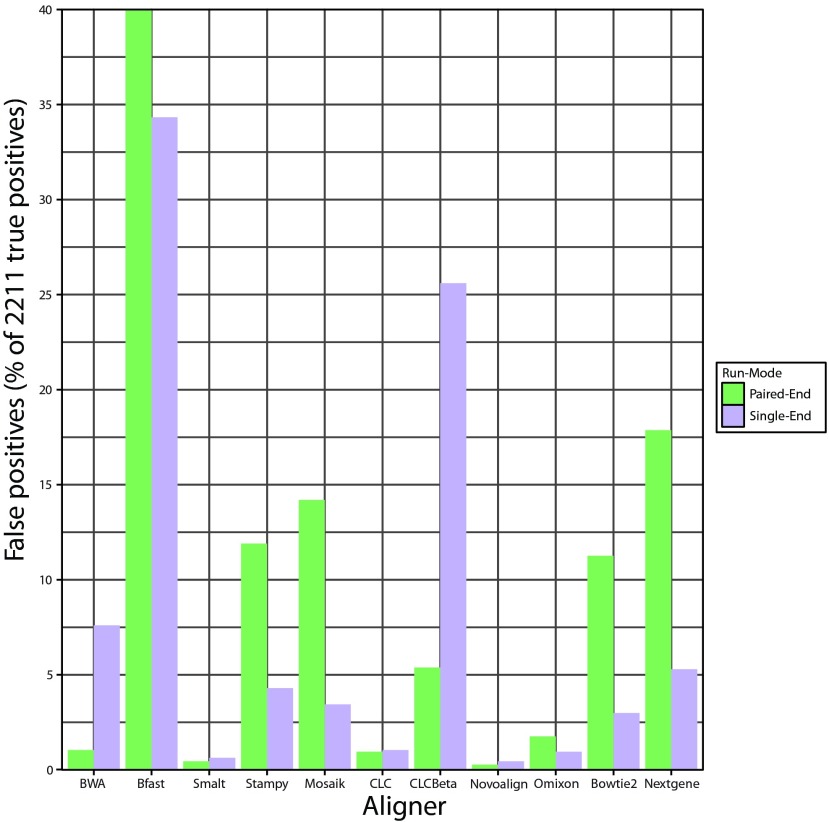
Graphical representation of the total number of downstream false positives expressed as a percentage of the true positive mutations detected following alignment with each aligner in single and paired-end mode across 67 groups of simulated Illumina reads. Each read group contained 20 SNPs and 13 INDELs.

Controlling the rate of false positive results is clinically important in avoiding unnecessary treatment, expense and patient anxiety. The number of incorrectly identified mutations varied widely dependent on aligner and run-mode ( Figure 2, Table 5). The highest downstream false-positive rates were obtained after alignment with Bfast, followed by CLC Bio’s Beta aligner, Nextgene, Mosaik, Stampy, and Bowtie 2. Novoalign and Smalt had the lowest downstream false-positive rates followed closely by Omixon and CLC. Number of false positives alone is of limited utility in assessing aligner performance, however positive predictive value (PPV) provides a useful metric which combines counts of both true and false positives in a single value ( Table 6). PPV was calculated based on the equation:

$$\text{PPV}=\frac{\text{True Positives}}{\text{True Positives}+\text{False Positives}}$$

**Table 5.** Raw numbers of false positive and negative mutations detected across 67 groups of simulated Illumina reads following alignment with each aligner in single and paired-end mode. Each read group contained 20 SNPs and 13 INDELs.

Aligner	# False positives PE	# False positives SE	# False negatives PE	# False negatives SE
BWA	23	168	17	121
Bfast	884	759	206	269
Smalt	10	14	32	33
Stampy	263	95	12	21
Mosaik	314	76	65	109
CLC	21	23	44	46
CLCBeta	119	566	69	72
Novoalign	6	10	9	22
Omixon	39	21	13	19
Bowtie2	249	66	24	40
Nextgene	395	117	30	29

**Table 6.** Positive predictive values (PPVs) calculated for each aligner in single and paired-end mode based upon downstream detection of mutations across 67 groups of simulated Illumina reads each containing 20 SNPs and 13 INDELs. In this case PPV is the total number of true positives divided by the sum of the total number of true positives and true negatives.

Aligner	PPV PE	PPV SE
BWA	98.96	92.56
Bfast	69.40	71.90
Smalt	99.54	99.36
Stampy	89.32	95.84
Mosaik	87.24	96.51
CLC	99.04	98.95
CLCBeta	94.74	79.08
Novoalign	99.73	99.55
Omixon	98.26	99.05
Bowtie2	89.78	97.05
Nextgene	84.67	94.91

No inferences were made about prevalence in calculating the values. Novoalign achieved the highest downstream PPV in both single and paired-end mode. Smalt, CLC and Omixon’s Variant Toolkit also performed strongly on this metric. Notably Stampy performed relatively poorly in contrast to the high downstream detection sensitivity it achieved.

### Paired-end vs. single-end reads

Notably Bfast, Nextgene, Stampy, Mosaik and Bowtie 2 all showed obvious increases in the number of downstream false positives detected following paired-end alignment vs single-end alignment. Conversely, switching from paired-end to single-end reads had varying adverse effects on the downstream detection sensitivities for all aligners except for Nextgene. The worst affected was BWA which retained all downstream SNP calls but lost 13% of INDEL calls. Notably Nextgene and Omixon’s Variant Toolkit were the only software to retain all INDEL calls when switching from paired to single-end mode. Omixon’s Variant toolkit achieved the highest sensitivity of dowsntream SNP and INDEL detection in single end mode with a 99.14% overall detection sensitivity.

This strong performance in single-end mode is relevant not only from a diagnostic standpoint, but also from a clinical cost-saving perspective as paired-end protocols ultimately incur extra costs per run vs single-end protocols. While paired-end reads generally represent a saving in terms of cost per megabase, they effectively double sequencing output and this may not be a cost-effective option depending on the logistics of the individual run. Furthermore, researchers who outsource sequencing will often see the available protocol for their relatively small sequencing project dictated by the larger projects they are multiplexed alongside.

### Effect of INDEL size on detection

**Figure 3.**
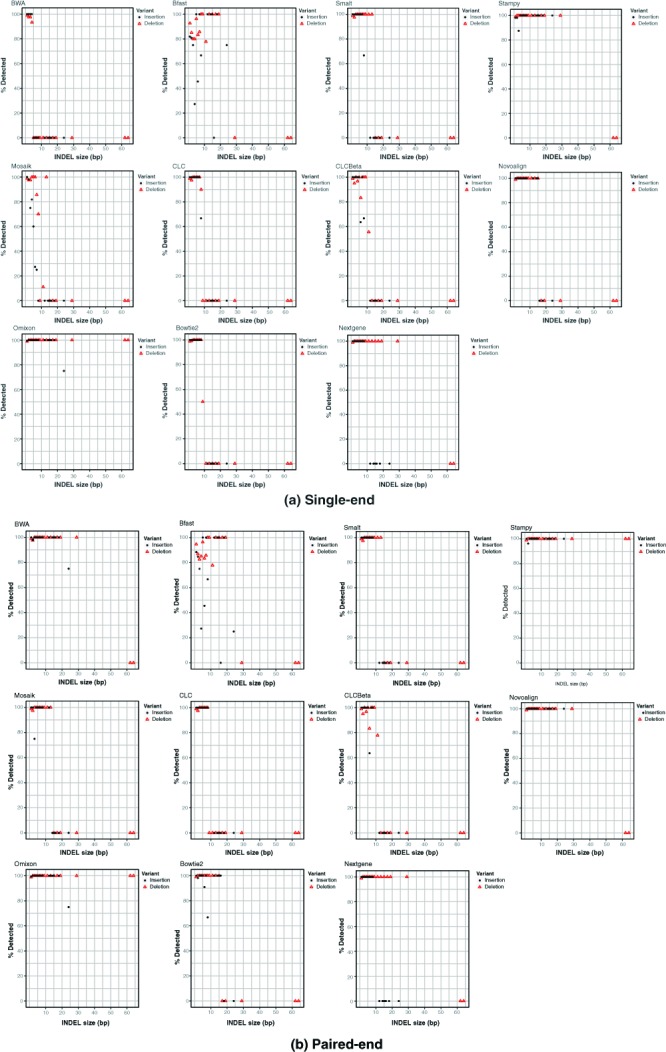
Effects of INDEL size on success of detection for all aligners in single-end (top panel) and paired-end (bottom panel) mode across 67 groups of simulated Illumina reads, each containing 13 INDELs of varying sizes.

Ability to facilitate downstream calling of INDELs varied by aligner with INDEL sizes influencing detectability in most cases ( Figure 3). The effect of size varied by aligner and run-mode with BWA and Novoalign enabling good downstream detection rates for all but the two largest deletions in paired-end mode while others such as Bowtie 2 and the CLC aligners didn’t achieve downstream detection far beyond a 10bp INDEL size. Stampy and Omixon eanbled good downstream detectability across the range of mutation sizes in the panel. Run-mode clearly affected detection of INDELs in some instances. For example BWA saw a marked decrease in downstream INDEL calling for single-end mode compared to paired-end while the Omixon results appeared unaffected by switching run-modes. Downstream detection varied between insertions and deletions also. For example, Nextgene enabled higher downstream sensitivity for insertions than deletions. Stampy and Omixon’s Variant Toolkit were the only aligners that enabled detection of the two largest deletions in the mutation panel. Collectively these observations highlight a need for those involved with testing and analysis to develop an appreciation of the various mutations that might exist in their target genes and to select their analysis tools appropriately. INDELs in the range represented by the *BRCA1* mutation panel have real-world relevance in genetic disorders and strong aligner performance on larger INDELs appears to be an exception rather than a rule.

## Summary and conclusions

Using a simulated, targeted sequencing scenario with Illumina read data, the work presented here highlights several important considerations regarding aligner choice in studies involving profiling of mutations. Furthermore the data presented goes some way to characterizing the performance of a comprehensive selection of commonly used aligners and should represent a useful resource for anyone focused on similar scientific studies.

Whilst the dataset used in this study was engineered to include a challenging range of mutations and efforts were made to simulate the error profile of Illumina sequencing technology, it is nevertheless a simplified representation of real-world data. Experimental artifacts such as PCR stutter have the potential to present further challenges to alignment algorithms and there is no consideration of such issues here. Furthermore, the test dataset used in this study produced a uniform, high coverage of a single target gene and only homozygous variants were simulated. Finally, the use of only a single variant-caller in the study means that some of the errors encountered may not be due to alignment issues. The aim is to follow up the current study by focusing on an expanded gene-set, alternative variant-callers, homo and heterozygous mutations and different sequence formats. Nonetheless, this focused study demonstrates the utility of simulated data in assessing program performance.

With the exception of Bfast, all aligners performed relatively well on the *BRCA1* dataset. Clinical applications necessitate the use of the most highly accurate solutions, however. Only Novoalign, Omixon’s Variant Toolkit and Stampy enabled 99% or greater sensitivity in both paired-end and single-end modes. Omixon and Stampy were the only two aligners to facilitate detection of the longest deletions in the dataset however Stampy’s performance was let down by a downstream false positive rate which would be considered unacceptably high for many applications. While Novoalign did not enable detection of the largest deletions in the test dataset, it was the most sensitive in paired-end mode and performed fastest on this dataset. Nevertheless, assuming the longer run-times are not an issue, Omixon’s superior sensitivity in single-end read mode likely makes it the best option when paired-end protocols are not possible. While the tests here produce some clear winners, they also serve to highlight that program performance can vary widely based on the fine-details of a particular run. Even the strongest overall performer can be found lacking in some respects and this means that researchers should be vigilant in their selection of tools for a particular application. In certain instances it may even be necessary to combine two or more approaches to ensure that all relevant aspects of a given dataset are sufficiently characterized and any approach will still require some level of visual inspection and quality control in a clinical setting. The data presented here should facilitate and expedite selection of the correct aligner for a particular task but they do not obviate the requirement for careful consideration nor further testing and analysis on the part of the end-user.
