# Supplementary material for: Considerations for clinical read alignment and mutational profiling using next-generation sequencing
Source: F1000Res. 2012 Sep 20;1:2. Originally published 2012 Jul 16. [Version 2] doi: 10.12688/f1000research.1-2.v2 (PMC3945748; doi:10.12688/f1000research.1-2.v2)
Supplement: Peer review history

# Peer review: Referee response for version 2

**Published:** 2012-10-29

**Authors:** Mihaela Pertea, Steven Salzberg

**Affiliations:** 1 McKusick-Nathans Institute of Genetic Medicine, Johns Hopkins University School of Medicine, Baltimore, MD, USA

Our ‘Not Approved’ status still maintains. It seems like he has made some nice improvements but the paper doesn’t address our fundamental concern that, despite its claims, it doesn’t evaluate aligners, but their capacity to work with the GATK pipeline. All the article really shows is how a particular program, GATK, functions in concert with different aligners. GATK has been fine-tuned to use BWA, as its own developers acknowledge. In our opinion these findings are misleading.

We have read this submission. We believe that we have an appropriate level of expertise to state that we do not consider it to be of an acceptable scientific standard, for reasons outlined above.

# Peer review: Referee response for version 1

**Published:** 2012-07-27

**Authors:** Mihaela Pertea, Steven Salzberg

**Affiliations:** 1 McKusick-Nathans Institute of Genetic Medicine, Johns Hopkins University School of Medicine, Baltimore, MD, USA

In this paper the author sets out to investigate the performance of several alignment tools and to assess their ability to accurately detect known mutations when used in a variant calling pipeline. This is an important issue to address before designing a particular analysis pipeline for variant detection. However, this paper makes multiple very strong claims about the superiority of various alignment algorithms based on highly flawed computational experiments. Overall the results are at best misleading, and many of the conclusions are simply wrong.

Our concerns are related to the following issues:

**1. Concerns about the experimental design:**

The experiment claims to measure the accuracy, and in particular the sensitivity and FDR rate, for many sequence aligners. Unfortunately, it simply doesn’t measure anything of the sort. Instead, it measures the sensitivity and FDR of the GATK SNP pipeline, a complex series of programs with many, many parameters, with different aligners fed into the very first step of GATK. GATK is exquisitely sensitive to these parameters; in our experience we can easily increase the number of SNPs by a factor of 5-fold simply by varying its parameters, REGARDLESS of the alignments provided at the front end. Unless the author optimizes GATK for each aligner – which he explicitly did not do – these results are simply invalid. Thus the whole experiment is deeply flawed.

It is not sufficient, in a benchmarking test like this one, to use only default running parameters (as the author says he did), and to make no effort at careful evaluation of what would be the best parameters to use for each aligner in that specific experiment. If the author wishes to compare aligners as part of a complex pipeline (GATK), he needs to do much more work than the simple push button runs he did here.

The whole point of simulated data ought to be that one can check each read and see if it was aligned to the correct place. This should be easy to do as all the reads are simulated and therefore their location is known a priori. If (and only if) the author compared the alignments to the true alignment, then he could report valid findings about the sensitivity at finding SNPs, indels, etc. He did not do this, which is somewhat astonishing. As it stands, the main results – including Tables 3 and 4 and Figure 1 – are simply wrong.

Also, in order to make his results reproducible, the author should provide the alignment results for all programs, as well as the exact command lines used for each aligner. Just specifying that he ran the aligners with parameters “as close as possible” to defaults is not enough.

**2. Running time evaluations:**

Another major conclusion of the paper concerns run times, which the author reports in a separate section (3.2). An obvious flaw here is that running the aligners on such small datasets (each only 200,000 reads) cannot properly differentiate the relative running times of the different programs, especially the faster ones. Exome sequencing, a very common experiment today, generates roughly 100 million reads per experiment – 500 times larger than each sample data used here. Whole-genome data sets are much larger. To provide any realistic run time findings, the author needs to load at least an exome-sized data set and run it. He doesn’t need to use simulated reads – many exomes are publicly available. Since he is only measuring run time, he doesn’t need to worry about the sensitivity of these alignments, just speed.

If the author wants to report findings about run-time, he needs to scrap this experiment and run a more realistic data set. If 100 million reads, not large by today’s standards, swamps the ability of any aligner to handle it, then he can report that.

Other comments in the alignment section are not justified. For example, claiming that “most alignment times recorded here might be considered manageable for most purposes” seems to be little more than the author’s unsupported opinion, based on a relatively tiny number of reads.

**3. Other significant concerns:**

a). The author used the Stampy package to simulate the reads from the BRCA1 region. What was the reason that this particular read simulator was used, and not another one that is independent from all aligners involved? E.g., the Mason simulator is considered to be relatively realistic. The Stampy simulator might give an unfair advantage to the Stampy aligner.

b). Why did the author align the simulated reads only to chromosome 17? If this is supposed to simulate a targeted sequencing experiment, why not just align to the BRCA1 region, which is far, far smaller than the entire chromosome? A much more realistic design would be to align to the whole human genome, which is normally done for real data where contamination from other parts of the genome is common. The author should also specify how he obtained the index required by the different aligners, and how long it took to create such an index (from the running times of the programs presented in the paper I assume this time was not included).

c). The way the programs were run is completely unclear, since no command line options are provided. Besides a step required to create an index (see above), some of the aligners require two steps to be run (e.g. BWA requires both an ‘aln’ and a ‘samse/sampe’ commands to be run; Stampy can be run in a hybrid version with a BWA option first). Were both of these steps included in the running times presented? Most of these programs have many options that can increase their sensitivity at the cost (sometimes small, sometimes not) of increased run time.

d). The author makes a technical error in classifying aligners into two categories, “based on either hash tables or suffix trees.” The Burrows-Wheeler Transform (the basis of Bowtie, BWA, and SOAP2) is simply not a suffix tree. Further, it is not only simplistic but incorrect to state that hash-based programs are generally more sensitive, while the ones based on suffix trees are faster. That is wrong in multiple ways; there are many examples of hash-based approaches that are fast but not sensitive, and suffix-tree approaches don’t have to be faster. These features (speed/sensitivity) depend much more on the numerous implementation details, of which the author appears to be unaware.

e). The two wrapper scripts (sam2bam.sh and gatk.sh) that the author mentions that he made available do not seem to be present.

f). Each of the 67 data sets presented in the paper include 20 SNPs and 13 indels. Why use 67 data sets? And why have exactly the same number of SNPs and indels in each one? What criteria were used to include these particular numbers of SNPs and indels? Since each data set is representative for only one variant of the BRCA1 gene, how likely it is that in real data these 20 SNPs and 13 indels will appear at the same time in the gene? This is an unrealistic data set that has a bizarrely skewed bias.

g). The author states – when referring to Figure 3 – that the size of the indels influences their detection rates. He specifically says that the “size of the effect varied by aligner with BWA and Novoalign showing good detection rates for all but the largest deletions.” This statement is simply not correct: BWA cannot find large deletions (by design). Neither can Bowtie. However, GATK can find larger deletions in some cases, even if the input alignments don’t detect them. There are also entirely separate programs (e.g., Pindel) designed to find larger indels, and researchers looking for large indels know about these programs (and use them). This whole discussion again reflects the fundamental flaw in the experimental design: the author is measuring GATK’s performance, not the performance of the aligners. In addition, the author’s interpretation of Figure 3 seems biased, and is not supported by the data in the figure itself.

**4. Minor concerns:**

a). PPV is defined differently in the main body of the paper and in Table 6′s caption.

b). The author needs to include citations or at least web addresses for all the aligners presented in the paper.

c). I assume GLG in Table 5 is in fact CLC.

d). Where did the author collect the known mutations for the BRCA1 gene from? He needs to provide citations.

We have read this submission. We believe that we have an appropriate level of expertise to state that we do not consider it to be of an acceptable scientific standard, for reasons outlined above.

# Peer review: Referee response for version 1

**Published:** 2012-07-26

**Authors:** Vera Kalscheuer

**Affiliations:** 1 Department of Human Molecular Genetics, Max Planck Institute for Molecular Genetics, Berlin, Germany

I have read this submission. I believe that I have an appropriate level of expertise to confirm that it is of an acceptable scientific standard.

# Peer review: Referee response for version 1

**Published:** 2012-07-25

**Authors:** Thomas Friedman

**Affiliations:** 1 Laboratory of Molecular Genetics, National Institute on Deafness and Other Communication Disorders (NIDCD), National Institutes of Health, Rockville, MD, USA

I have read this submission. I believe that I have an appropriate level of expertise to confirm that it is of an acceptable scientific standard.
